# Culturable bacteria associated with *Anopheles darlingi* and their paratransgenesis potential

**DOI:** 10.1186/s12936-020-03574-1

**Published:** 2021-01-13

**Authors:** Elerson Matos Rocha, Osvaldo Marinotti, Deidre Machado Serrão, Laura Viana Correa, Ricardo de Melo Katak, Campos-de-Oliveir Juan, Veranilce Alves Muniz, Marta Rodrigues de Oliveira, Joaquim Ferreira, Marcos Cézar Fernandes Pessoa, Rosemary Aparecida Roque, Adolfo Jose da Mota, Piero Onorati, Jayme A. Souza-Neto, Olle Terenius, Wanderli Pedro Tadei

**Affiliations:** 1 Programa de Pós-Graduação em Biotecnologia, Universidade Federal do Amazonas - PPGBIOTEC / UFAM, Manaus, Brazil; 2 MTEKPrime, Aliso Viejo, CA USA; 3 Universidade Estadual do Amazonas - MBT, UEA, Manaus, Brazil; 4 Universidade Estadual do Amazonas - BIONORTE, UEA, Manaus, Brazil; 5 Laboratório de Malária E Dengue, Instituto Nacional de Pesquisas da Amazônia, INPA, Manaus, Brazil; 6 Department of Ecology, Swedish University of Agricultural Sciences (SLU), Box 7044, 750 07 Uppsala, Sweden; 7 School of Agricultural Sciences, Department of Bioprocesses and Biotechnology, Central Multi User Laboratory, São Paulo State University (UNESP), Botucatu, Brazil; 8 Department of Cell and Molecular Biology, Microbiology, Uppsala University, Box 596, 751 24 Uppsala, Sweden

**Keywords:** Mosquito, Malaria, Microbiota, Vector-borne disease, Amazon forest

## Abstract

**Background:**

Malaria remains a major public health problem in South America, mostly in the Amazon region. Among newly proposed ways of controlling malaria transmission to humans, paratransgenesis is a promising alternative. Paratransgenesis aims to inhibit the development of parasites within the vector through the action of genetically modified bacteria. The first step towards successful paratransgenesis in the Amazon is the identification of *Anopheles darlingi* symbiotic bacteria, which are transmitted vertically among mosquitoes, and are not pathogenic to humans.

**Methods:**

Culturable bacteria associated with *An. darlingi* and their breeding sites were isolated by conventional microbiological techniques. Isolated strains were transformed with a GFP expressing plasmid, pSPT-1-GFP, and reintroduced in mosquitoes by feeding. Their survival and persistence in the next generation was assessed by the isolation of fluorescent bacteria from eggs, larvae, pupae and adult homogenates.

**Results:**

A total of 179 bacterial strains were isolated from samples from two locations, Coari and Manaus. The predominant genera identified in this study were *Acinetobacter*, *Enterobacter*, *Klebsiella*, *Serratia*, *Bacillus*, *Elizabethkingia*, *Stenotrophomonas* and *Pantoea*. Two isolated strains, *Serratia*-Adu40 and *Pantoea*-Ovo3, were successfully transformed with the pSPT-1-GFP plasmid and expressed GFP. The fluorescent bacteria fed to adult females were transferred to their eggs, which persisted in larvae and throughout metamorphosis, and were detected in adult mosquitoes of the next generation.

**Conclusion:**

*Serratia*-Adu40 and *Pantoea*-Ovo3 are promising candidates for paratransgenesis in *An. darlingi.* Further research is needed to determine if these bacteria are vertically transferred in nature.

## Background

Malaria remains a major public health problem worldwide, with more than 200 million cases and nearly half a million deaths annually. Prompt diagnosis and treatment, preventive therapy and vector control are tools presently available to prevent malaria disease and death [1]. Despite substantial progress toward malaria control and elimination in the Americas, malaria has seen a resurgence in the last years due to political unrest, worsening social and economic crisis and large-scale migration [2].

*Anopheles darlingi* is a major malaria vector in South America [1, 3, 4] and, therefore, a major target of vector control. Its importance as a malaria vector spurred studies of *An. darlingi* biology [5–7], behaviour [8, 9], physiology and biochemistry [10], genetics [11–13], and insecticide resistance [14, 15]. Because *An. darlingi* is anthropophilic, opportunistic, exophagic, and exophilic, long-lasting insecticidal nets and indoor residual spraying are not effective alone and novel vector control methods are needed to improve the control of malaria transmission by these mosquitoes [16–18]. For example, strategies deploying genetically-modified mosquitoes [19, 20] and their endosymbionts (paratransgenesis) [21–24] have been proposed as vector-based tools for malaria control.

Paratransgenesis entails the colonization of mosquito gut with genetically engineered bacteria that are effective in inhibiting parasite development. Huang et al*.* [23] proposed that ideal endosymbionts for effective paratransgenesis application are easily manipulated genetically, colonize mosquitoes efficiently, spreading into mosquito populations (vertical and horizontal transmission), and are efficient in inhibiting pathogen development in mosquitoes. Proof-of-principle experiments such as those conducted by Yoshida et al*.* [25] and Wang et al. [26, 27] demonstrated that genetically modified bacteria are capable of interfering or blocking malaria parasite development in mosquitoes. Among the symbiotic bacteria found in malaria vectors, species belonging to the genera *Asaia*, *Pantoea*, *Serratia*, *Pseudomonas* and *Thorsellia* have been evaluated as candidates for paratransgenesis [28–30].

The microbial flora associated with *An. darlingi* has been investigated [31–36]. Here, in order to move forward toward paratransgenesis, symbiotic bacterial strains that are amenable to genetic manipulation, able to colonize *An. darlingi,* and are transferred from adult females to their progeny were identified.

## Methods

### Field collection of *Anopheles darlingi*

**Fig. 1:**
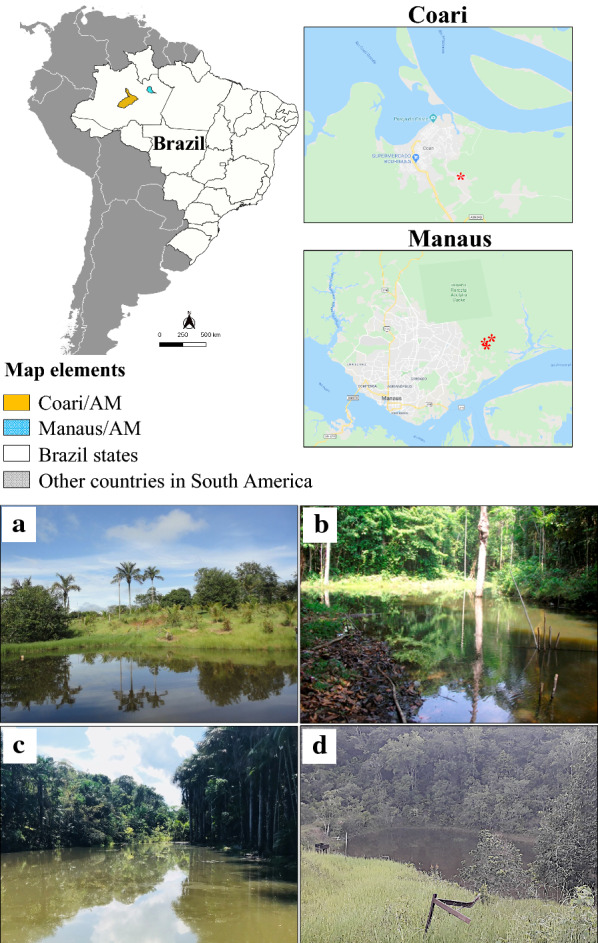
Sample collection sites: Top—Maps of locations where samples were collected. Red dots indicate the locations where insects and water were collected Bottom—Pictures of collection sites. **a** Coari—Itapeuá -Sítio do Gordo 4°06′45.5"S, 63°07′44.0"W; **b** Manaus, Puraquequara—Portela 3°02′47.0"S, 59°52′54.4"W: **c** Manaus, Puraquequara—Dona Chagas 3°02′33.5"S, 59°53′15.6"W: **d** Manaus, Brasileirinho—Raifram 3°02′11.2"S, 59°52′17.4"W

Adults, pupae, and larvae of *An. darlingi* and water from their breeding sites were collected in Coari and Manaus (Table 1, Fig. 1). These are areas of active malaria transmission, as determined by the Vigilância Epidemiológica da Secretaria Municipal de Saúde de Manaus (Epidemiological Surveillance of the Municipal Health Secretariat in Manaus). Mosquito collections were performed in a single site in each location, for three hours during dusk.

**Table 1:** Geographic location and characteristics of the *Anopheles darlingi* breeding sites where water, larvae and pupae were collected. Adults were captured in the same locations

Local	Description	GPS coordinates	Date
Coari—Itapeuá -Sítio do Gordo	Semirural/Dug ponds/ fish farming	4°06′45.5"S, 63°07′44.0"W	01/2014
Manaus, Puraquequara—Portela	Semirural/Natural lake with small fish	3°02′47.0"S, 59°52′54.4"W	02/2017
Manaus, Puraquequara—Dona Chagas	Semirural/Natural dam/fish farming	3°02′33.5"S, 59°53′15.6"W	02/2018
Manaus, Brasileirinho—Raifram	Semirural/Private dam/ fish farming	3°02′11.2"S, 59°52′17.4"W	02/2018

Surface microlayer (SML) water samples were collected using a stainless-steel mesh screen measuring 400 cm^2^, with a mesh size of 1.25 × 0.36 mm wire diameter as described previously [37]. Water samples were transferred to sterile 50 ml tubes and stored on ice.

Mosquito larvae and pupae were collected with a 350 ml aluminum dipper, using standard dipping techniques [38]. Larvae and pupae were transferred to sterile plastic containers containing sterile water and stored on ice. Adult mosquitoes were collected during dusk in peri-domicile and extra-domicile environments. Adult females were captured by human landing catches (HLC), by trained technicians using personal protective equipment. Only non-blood fed females were selected for this study. Captured mosquitoes were transferred to 300 ml wax-coated paper cups and transported to the laboratory. Water, larvae, pupae and adult mosquito samples were transported to the laboratory and processed within 24 h. No mosquito mortality occurred during transportation. Species were identified using morphological characters [39].

Mosquito husbandry was at 29 °C, 80% relative humidity and 12/12 light/dark cycles. Larvae were fed autoclaved fish food (Tetramin). Adults were given access to 10% sucrose solution ad libitum and fed on chicken blood when required for the experiments described below. All the protocols used in these studies received ethical clearance from the Brazilian National Institute for Amazonian Research (INPA) Ethics Committee and the biological material analyzed during the present study was collected with official collecting permission (17524-1 and 21264-5) given by ‘Sistema de Autorização e Informação em Biodiversidade’ (SISBIO) of the Brazilian Ministry of Environment (MMA).

### Bacteria isolation and morphological characterization

Ten larvae, ten pupae and ten adults from each collection site were selected for bacterial isolation. Breeding water samples were plated directly from the transportation tubes, 50 µl per Petri dish. *Anopheles darlingi* larvae (3rd and 4th instar), pupae, adult females and pools of 10 eggs were sequentially immersed five times, for a few seconds each time, in 70% ethanol and then deionized, autoclaved sterile water before being homogenized. Each larva, pupa, adult or eggs pool were individually homogenized in 1 ml sterile water and centrifuged for 3 min at 8,000 rpm. The pellet was resuspended in 200 µl of sterile water by vortexing and centrifuged again for 1 min at 800 rpm. From the supernatant, 50 µl were plated in each Petri dish and spread with a Drigalski spatula. Bacteria extracted from all samples were plated on 90 mm Petri dishes containing Tryptic Soy Agar (TSA), Nutrient Agar (NA) or Luria–Bertani Agar (LB) medium. Fluconazole (20 mg/ml) was added to inhibit fungal growth.

Cultures were incubated at 29 °C for 24 h. Negative control plates with only sterile water resulted in no colonies. The streak plate technique was applied for isolating specific bacteria from the original colonies potentially containing a mixture of microorganisms.

Colony morphology was inspected for size, shape, texture, elevation, and color. Additionally, cell type and Gram staining were examined for each isolate using standard microbiological techniques and a magnification microscope. Each isolate was preserved in 2 ml cryotubes containing Nutrient Broth, 20% glycerol at − 80 °C. Colonies displaying similar morphological and staining characteristics were assigned to groups and three representatives from each group were randomly selected for 16S rRNA gene sequencing.

### DNA extraction, 16S rRNA gene amplification by PCR and sequence analyses

DNA extraction, from isolated bacterial colonies, was performed with InstaGene™ Matrix (BioRad) following the manufacturer’s instructions. DNA was spectrophotometrically quantified and adjusted to 150 nanograms/µl. Bacterial 16S rRNA genes were amplified by PCR using Taq Pol—Master mix 2× (Cellco Biotec), and the primers 27F (5′-AGAGTTTGATCMTGGCTCAG-3′) [40] and 1100R (5′-AGGGTTGCGCTCGTT-3′) modified from Sawada et al*.* [41] used in a previous study [31]. Each reaction consisted of 12.5 µl Master mix; 1 µl DNA (150 ng/µl); 10.5 µl H_2_O milli-Q and 0.5 µl (10 pMol) of each primer. The PCR program had an initial denaturation at 95 °C for 3 min, followed by 35 cycles of [94 °C for 1 min, 54 °C for 40 s, 72 °C for 90 s], followed by a final extension at 72 °C for 5 min. Amplicon production and size were verified by electrophoresis in a 0.8% agarose gel, stained with ethidium bromide. Amplicons were purified with PCR Purification Kit (Cellco Biotec), following the manufacturer’s instructions and 200 ng of purified DNA was used for each sequencing reaction (BigDye Terminator V 3.1., Life Technologies and 10 pMol of primer). 27F and 1100R primers were used in separate sequencing reactions, generating data from both DNA strands.

Sequences were assembled using CAP3 program with the capability to clip 5′ and 3′ low-quality regions of reads, apply quality values in overlaps between reads, and generate consensus sequences [42]. Taxonomic assignments of the isolated bacteria were based on comparisons of the consensus sequence with 16S sequences in GenBank applying BLASTn (https://blast.ncbi.nlm.nih.gov/Blast.cgi) at the National Center for Biotechnology Information (NCBI) and the Ribosomal Database Project, RDP-II (http://rdp.cme.msu.edu/comparison/comp.jsp).

### Bacterial transformation

Bacterial strains isolated in this study were grown in Nutrient broth. Electrocompetent cells were then prepared as described by Gonzales et al*.* [43] and transformed with the plasmid pSPT-1-GFP using an Electroporator 2510 (Eppendorf®). The plasmid pSPT-1-GFP (Additional file 1: Document S1) was kindly provided by Dr. Spartaco Astolfi Filho (CAM-UFAM). Following electroporation, bacteria were plated on Nutrient Agar containing 100 mg/ml ampicillin and incubated for 16 h at 37 ºC. Colonies expressing green fluorescent protein were identified by exposure to UV light, and were selected for further studies.

### Vertical transmission

All experiments were conducted in three biological replicates. Wild *An. darlingi* females were captured in Manaus, Raifram (Table 1) and transferred to the laboratory and kept without access to food and water for 12 h. In each replica, 50 females were separated in three plastic cups (20, 20 and 10). The cup containing 10 mosquitoes (Control) was fed with cotton balls soaked in 10% sucrose for 6 h. Sterile cotton balls soaked in 10% sucrose containing 100 mg/ml ampicillin and ~ 3 × 10^4^ CFU/ml of GFP expressing bacteria (*Serratia*-Adu40 or *Pantoea*-Ovo3) were placed on the top of the mosquito cages containing 20 mosquitoes for 6 h. Then, adult females were fed on chicken blood for 1 h and kept in the insectary until oviposition. Eggs were collected on moist sterile filter paper containing 100 mg/ml ampicillin.

The exterior of ten eggs from each female was surface-rinsed with 70% ethanol for 1 min, then washed for 1 min in sterile distilled water. Surface-rinsed eggs were immediately homogenized and plated on Nutrient Agar plates with 100 mg/ml ampicillin. After oviposition, females were treated with 70% ethanol and sterile water, as described for the eggs. Their whole bodies were homogenized and plated on Nutrient Agar plates with 100 mg/ml ampicillin. The remaining eggs were treated similarly for surface-rinsing with 70% ethanol and transferred to plastic containers filled with sterile deionized water containing 100 mg/ml ampicillin and food for development of the F1 generation. The containers were sterilized daily, water was discarded and replaced with fresh sterile water containing 100 mg/ml ampicillin and food. Ten larvae from each developmental instar (1st, 2nd, 3rd and 4th), 10 pupae and 10 three-day-old, sugar fed adults emerging from each family were rinsed with 70% ethanol and sterile water, as described above. Their whole bodies were homogenized and plated on Nutrient Agar plates with 100 mg/ml ampicillin. Bacterial colonies displaying GFP expression were identified by exposure to UV light after incubation for 16 h at 37 ºC.

One set of experiments similar to that described above, but omitting ampicillin during the development of F1 mosquitoes, was performed to evaluate the effect of the antibiotic on the survival and persistence of GFP positive bacteria in *An. darlingi*.

## Results

### Bacteria isolation and species identification

A total of 900 bacterial colonies were included in this study, which were organized in 126 groups of morphologically similar colonies. Bacteria were isolated from *An. darlingi* samples collected in two municipalities, Manaus and Coari, including larvae, pupae, adult females, their breeding site water and *An. darlingi* eggs. There was no apparent difference in the number of colonies formed when plating breeding water and mosquito extracts in three growing media (TSA, NA and LB), therefore, Nutrient agar and Nutrient broth were used to isolate pure cultures and establish stable frozen stocks. Three representative isolates from 84 randomly selected groups were further characterized by 16S rRNA gene PCR amplification and sequencing, resulting in 179 high quality sequences (Table 2).

**Table 2:** Isolated bacterial strains from Manaus and Coari included in each step of this work

	Isolates (purified colonies)	Groups (similar morphological characteristics)	Groups selected for 16S sequencing (random selection)	High quality 16S sequences
Manaus	722	105	68	155
Coari	178	21	16	24
Total	900	126	84	179

Blastn with the 179 high-quality 16S rRNA sequences revealed 35 genera of bacteria (Table 3). All sequences were registered in SisGen database (Sistema Nacional de Gestão do Patrimônio Genético e do Conhecimento Tradicional Associado) with the number A0F653E and are available in GenBank with accession numbers MT052372-MT052395 and MN709221-MN709375 (Additional file 1: Tables S1–S5).

**Table 3:** Bacteria isolated from *Anopheles darlingi* and natural breeding water

Genus	Eggs	Larvae	Pupae	Adults	Water
*Achromobacter*				C61 C64	
*Acinetobacter*	Ovo9 Ovo14	Lar11 C41 Lar3 Lar6 Lar15 Lar19 Lar27	Pup5 Pup14 Pup19	Adu2 Adu22 Adu29 Adu6 Adu16 Adu21 Adu44 Adu48 Adu36	Wat25 Wat1 Wat3 Wat5 Wat15 Wat21 Wat27
*Aeromonas*			Pup4	Adu13	
*Aquitalea*	Ovo19			Adu45	
*Arthrobacter*				Adu34	
*Azospirillum*			Pup15		
*Bacillus*	Ovo20 Ovo5 Ovo11	Lar1 Lar2 Lar22 C39 Lar28	Pup2 Pup10 Pup1 Pup22 C21 Pup7 C45	C55 Adu39 Adu11 Adu25 Adu15 Adu43 Adu27 C46	C14 C4 C67 Wat10 Wat30 Wat29 Wat18 C2 C22
*Brevibacterium*		Lar20			
*Burkholderia*		Lar10			
*Chromobacterium*		Lar4 Lar7	Pup6	Adu7 Adu23 Adu1 Adu18 Adu28 Adu26 Adu37	Wat4 Wat16 Wat26 Wat12 Wat20 Wat22
*Citrobacter*	Ovo1				
*Cronobacter*		Lar26			
*Cupriavidus*	Ovo6			Adu8 Adu17	Wat34
*Elizabethkingia*	Ovo12	Lar21		Adu12 Adu42	
Enterobacter ***	Ovo2	Lar9 Lar16 Lar12 Lar24	Pup17	***Adu24*** Adu30 Adu49 Adu20	Wat6 Wat19 Wat13 Wat7
*Exiguobacterium*			Pup16		
*Flectobacillus*			Pup3		
*Herbaspirillum*				Adu31	
*Klebsiella*	Ovo7	Lar5 Lar25	Pup18 Pup20	Adu14 Adu47 Adu35	C30 Wat8 Wat14 Wat24 Wat33 Wat2 Wat28
*Leucobacter*			Pup8 Pup21		
*Lysinibacillus*	Ovo16			Adu41	
*Microbacterium*	Ovo13 Ovo17			Adu19	
*Moraxella*	Ovo8			Adu9	
*Nubsella*		Lar18			
*Paenibacillus*		Lar8			
Pantoea ***	***Ovo3*** Ovo15	Lar13	Pup12	Adu3 ***Adu38***	Wat32
*Pectobacterium*			Pup11		
*Pseudomonas*	Ovo10	Lar17		Adu5 Adu46 Adu10	Wat31
*Ralstonia*			Pup9		
*Rhizobium*					Wat17
Serratia ***	Ovo4	C19 C36 C37 Lar14 Lar23	Pup13 Pup23	C38 C58 ***Adu40***	C6 C9 Wat9 Wat23
*Siccibacter*					C5 C7 C29
*Sphingobacterium*	Ovo21				
*Staphylococcus*				Adu33	
*Stenotrophomonas*	Ovo18			Adu4 Adu32	Wat11
**35 unique genera of bacteria**	**179 Strains**				

Strains were isolated from larvae, pupae, adult female mosquitoes and breeding site water from Coari (C), or Manaus. Strains from Manaus are isolated from the *Anopheles darlingi* eggs (Ovo), Larvae (Lar), Pupae (Pup), adult females (Adu) or breeding site surface water (Wat), strains selected for transformation with the plasmid pSPT-1-GFP are indicated with *** and the strains transformed for additional studies are indicated in bold and underlined. GenBank accession numbers for the 16S ribosomal RNA gene sequences are shown in Additional file 1: Tables S1–S5

Five genera from Proteobacteria (*Achromobacter*; *Acinetobacter*; *Klebsiella*; *Serratia*; and *Siccibacter*) and one Firmicutes genus (*Bacillus*) were identified in samples from Coari. *Serratia* and *Bacillus* were the most common (Additional file 1: Table S1). Samples from Manaus included representatives of Proteobacteria; Firmicutes; Actinobacteria; and Bacteroidetes, distributed in 34 genera. The predominant genera were *Bacillus*, *Acinetobacter*, *Pseudomonas* and *Chryseobacterium*. Proteobacteria with the largest representation were *Acinetobacter*, *Enterobacter*, *Klebsiella*, *Serratia*, *Pantoea*, *Stenotrophomonas*, and *Klebsiella variicola* (Additional file 1: Table 2). Firmicutes species were also detected with *Bacillus* being the most abundant (Additional file 1: Table S3). Three genera of Bacteroidetes were detected with *Elizabethkingia* (4 strains) being the most abundant (Additional file 1: Table S4). Among four genera of Actinobacteria, *Microbacterium* and *Leucobacter* had three and two representative strains, respectively (Additional file 1: Table S5).

### Bacterial transformation for paratransgenesis

**Fig. 2:**
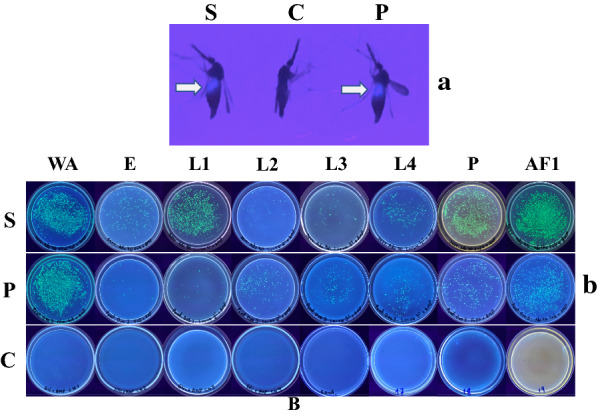
**a** Representative samples of fluorescent bacteria-fed mosquitoes. Adult female *An. darlingi* fed fluorescent bacteria *Serratia*-Adu40 (S) or *Pantoea*-Ovo3 (P), and control females not fed fluorescent bacteria (C). White arrows indicate fluorescent abdomen. **b** Fluorescent bacteria extracted from Wild females (WA) previously fed *Serratia*-Adu40 (S) or *Pantoea*-Ovo3 (P), and their progeny; eggs (E), Larvae 1st–4th instars (L1-L4), Pupae (P), and adult female F1 (A F1), in NA plates containing ampicillin. All experiments were conducted in triplicate with 10 eggs, 10 larvae, 10 pupae and 10 adult mosquitoes examined in each replicate. Each larvae, pupae, adult or eggs pool sample was homogenized in 1 ml sterile water and centrifuged for 3 min at 8,000 rpm. The pellet was resuspended in 200 µl of sterile water by vortexing and centrifuged again for 1 min at 800 rpm. From the supernatant, 50 µl were plated in each Petri dish. Fluorescent bacteria were detected in 100% of experimental samples analyzed and were never detected in control plates

Considering the frequency (number of isolated strains) and persistency (findings throughout development) of the identified bacterial strains observed in this study, as well as previous publications on mosquito paratransgenesis, the strains *Serratia*-Adu40*, Enterobacter*-Adu24, *Pantoea*-Adu38*,* and *Pantoea*-Ovo3, were selected for further investigation. *Serratia* and *Enterobacter* were detected in all developmental stages of *An. darlingi* as well as in the water of their breeding sites (Table 2). *Pantoea* species have been successfully applied for paratransgenesis studies in other mosquito species [23, 26]. Those four strains were submitted to electroporation with the plasmid pSPT-1-GFP; however, only *Serratia*-Adu40 and *Pantoea*-Ovo3 were amenable for transformation, as evidenced by producing green fluorescent colonies. To examine recombinant bacteria colonization and survival in the mosquito, female mosquitoes were fed GFP-expressing *Serratia* and *Pantoea* and their progeny examined for green fluorescent bacteria. Eggs, larvae, pupae and the next generation of adults were homogenized, and homogenates were plated on selective ampicillin-containing NA medium. For both mosquito groups fed GFP-*Serratia* and GFP-*Pantoea*, GFP-expressing bacteria were present in all individuals and developmental stages examined (Fig. 2). Similar results were obtained when F1 mosquitoes were maintained in the presence or absence of ampicillin. The 16S rRNA genes of GFP-expressing bacteria isolated from F1 mosquitoes (GenBank accession numbers MT102128 *Serratia-*Sm1 and MT102127 *Pantoea-*Pa1) are identical to those of the original *Serratia*-Adu40 and *Pantoea*-Ovo3 strains, indicating there was no horizontal transfer of the plasmid to other bacteria.

## Discussion

Identification of bacteria associated with vector mosquitoes has received particular attention since studies suggest that the vector gut microbiota affects the outcome of mosquito infection with *Plasmodium* parasites [44–46]. Additionally, paratransgenesis has been proposed as an alternative and supplemental approach for controlling malaria transmission by mosquitoes [21]. Paratransgenesis is based on the use of symbiotic bacteria to express anti-pathogen effector molecules inside the target vector species. For this approach, symbiotic bacteria are isolated, genetically modified to express anti-*Plasmodium* effector molecules, and then reintroduced into the mosquito, where they produce the desired effect [25, 26]. Therefore, ideal candidates for paratransgenesis are those easily manipulated genetically, which colonize mosquitoes efficiently, spreading into mosquito populations and are efficient in inhibiting pathogen development in mosquitoes [23].

Although studies have been carried out to identify the bacterial microbiota of malaria vector mosquitoes [30, 47–52], several important vector species remain un-studied or with limited information. Among those is *An. darlingi*, and other malaria vectors in the American continent. Here, the repertoire of culturable bacteria associated with *An. darlingi* was expanded and investigated for the potential for paratransgenesis. The predominant bacteria genera found associated with *An. darlingi* in this study, in decrescent number of isolated strains, were *Bacillus, Acinetobacter, Chromobacterium, Klebsiella, Serratia,* and *Enterobacter*. Other bacteria not amenable for isolation under our protocol likely complement the set of *An. darlingi* associated microorganisms. Some of those may be candidates for paratransgenesis in this mosquito species. For example, *Asaia* sp. was found associated with by Oliveira et al. [36] after amplification and sequencing of the V4 hypervariable region of the 16S rRNA. The ability of *Asaia* to colonize different species of malaria vectors and its demonstrated vertical and trans-stadial diffusion mechanisms have indicated its potential in paratransgenesis [29].

Colonies of *Acinetobacter*, *Bacillus*, *Enterobacter, Klebsiella, Pantoea* and *Serratia* were identified in samples extracted from all developmental stages of the mosquito and in their breeding sites, indicative of a strong association between these bacteria and *An. darlingi*. *Acinetobacter* and *Klebsiella* contain species which are potentially pathogenic [53, 54].

*Pantoea agglomerans* has been found in association with anopheline mosquitoes [31, 55]. Furthermore, Wang et al*.* [26] engineered *P. agglomerans* to secrete anti-malarial proteins in the mosquito midgut. Mosquitoes carrying these modified bacteria had a significant reduced infection prevalence and showed up to 98% inhibition of oocyst formation in mosquitoes fed an infectious meal.

Genetic transformation of four strains, *Serratia*-Adu40*, Enterobacter*-Adu24, *Pantoea*-Adu38*,* and *Pantoea*-Ovo3, was attempted with a plasmid that induces the expression of GFP, for the purpose of tracking bacteria transfer among mosquito developmental stages. However, successful identification of green fluorescent bacterial colonies was only achieved with *Serratia*-Adu40 and *Pantoea*-Ovo3. Additional effort for the transformation of mosquito-associated bacteria may include the optimization of methods for preparing transformation competent cells and the inclusion of other plasmids carrying different origins of replication and/or marker genes.

Mosquitoes were fed sugar meals containing either *Serratia-*GFP or *Pantoea-*GFP to examine recombinant bacteria colonization and survival in *An. darlingi* mosquitoes. Midguts of female mosquitoes that were fed GFP-tagged bacteria became strongly fluorescent, indicating that they were established and multiplying. Transstadial transfer of bacteria in *An. darlingi* was also investigated. Under the experimental conditions applied in this study, *Serratia-*GFP or *Pantoea-*GFP were transferred from gravid females to their eggs, persisted in the hatched larvae throughout all four instars, and remained for the duration of metamorphosis, to be detected in adults of the next generation. It is not clear how viable bacteria remained in eggs after surface-rinsing with 70% ethanol, since it has been demonstrated that vertical transmission occurs mostly by egg-smearing [56]. Possible contamination of eggs and other specimens following ethanol sterilization has been demonstrated previously and alternative methods of sterilization should be considered in future work [57, 58]. A significant number of bacteria was observed in pupae (Fig. 2), a surprising observation since an effective gut sterilization mechanism operating during mosquito metamorphosis has been reported [59]. Further investigation is needed to clarify these findings; however, whatever route of transmission occurred, vertical transmission was observed in this study.

Taken together, the results reported here indicate that it is possible to introduce genetically transformed bacteria in *An. darlingi* females by a sugar meal, that the bacteria may survive, multiply and express a GFP marker (eventually an anti-*Plasmodium* effector molecule), and that *Serratia*-Adu40 and *Pantoea*-Ovo3 are transferred from adult females to their eggs, from eggs to larvae and finally to adults in the next generation. Vertical transmission of *Serratia* sp. in *Anopheles* and *Pantoea* sp. in *Culex* mosquitoes has been demonstrated previously [60, 61].

Additional research is needed to determine if these bacteria are also vertically transferred in nature. Furthermore, careful studies need to be performed certifying that these isolated strains do not carry virulent genes causing pathologic conditions in humans, animals or plants.

While paratransgenesis technical tools may be available, implementing an effective and scalable paratransgenesis-based malaria control strategy is still in the conceptual stage. Issues related to efficacy and biosafety need to be addressed and a regulatory framework for this specific application needs to be established. However, inaction should not be the option. The potential public health benefit of new tools to reduce or even eradicate malaria is clear and widely recognized. The risks incurred by testing new, and unproven strategies and the risks to human health and the environment posed by maintaining the status quo should be taken into account in decision-making.

## Supplementary Information

**Additional file 1: Table S1. **Bacteria isolated from samples from Coari. **Table S2. **Proteobacteria isolated from samples from Manaus. **Table S3. **Firmicutes isolated from samples from Manaus. **Table S4. **Bacteroidetes isolated from samples from Manaus. **Table S5.** Actinobacteria isolated from samples from Manaus.

## Data Availability

All data generated or analysed during this study are included in this published article and its supplementary information files. DNA sequences are available at National Center for Biotechnology Information http://www.ncbi.nlm.nih.gov.

## References

[1] WHO. World malaria report. Geneva: World Health Organization; 2019. https://www.who.int/publications-detail-redirect/9789241565721

[2] Jaramillo-Ochoa R, Sippy R, Farrell DF, Cueva-Aponte C, Beltrán-Ayala E, Gonzaga JL (2019). Effects of political instability in Venezuela on malaria resurgence at Ecuador-Peru Border, 2018. Emerg Infect Dis.

[3] Tadei WP, Rodrigues IB, Rafael MS, Sampaio RTM, Mesquita HG, Pinheiro VCS (2017). Adaptative processes, control measures, genetic background, and resilience of malaria vectors and environmental changes in the Amazon region. Hydrobiologia.

[4] Carlos BC, Rona LDP, Christophides GK, Souza-Neto JA (2019). A comprehensive analysis of malaria transmission in Brazil. Pathog Glob Health.

[5] Charlwood JD (1996). Biological variation in *Anopheles darlingi* Root. Mem Inst Oswaldo Cruz.

[6] Vittor AY, Gilman RH, Tielsch J, Glass G, Shields T, Lozano WS (2006). The effect of deforestation on the human-biting rate of *Anopheles darlingi*, the primary vector of falciparum malaria in the Peruvian Amazon. Am J Trop Med Hyg.

[7] Hiwat H, Bretas G (2011). Ecology of *Anopheles darlingi* Root with respect to vector importance: a review. Parasit Vectors.

[8] Tadei WP, Thatcher BD, Santos JM, Scarpassa VM, Rodrigues IB, Rafael MS (1998). Ecologic observations on anopheline vectors of malaria in the Brazilian Amazon. Am J Trop Med Hyg.

[9] Marrelli MT, Honório NA, Flores-Mendoza C, Lourenco-de-Oliveira R, Marinotti O, Kloetzel JK (1999). Comparative susceptibility of two members of the Anopheles oswaldoi complex, *An. oswaldoi* and *An. konderi*, to infection by Plasmodium vivax. Trans R Soc Trop Med Hyg..

[10] Okuda K, Caroci A, Ribolla P, Marinotti O, de Bianchi AG, Bijovsky AT (2005). Morphological and enzymatic analysis of the midgut of *Anopheles darlingi* during blood digestion. J Insect Physiol.

[11] Scarpassa VM, Conn JE (2007). Population genetic structure of the major malaria vector *Anopheles darlingi* (Diptera: Culicidae) from the Brazilian Amazon, using microsatellite markers. Mem Inst Oswaldo Cruz.

[12] Mirabello L, Vineis JH, Yanoviak SP, Scarpassa VM, Póvoa MM, Padilla N (2008). Microsatellite data suggest significant population structure and differentiation within the malaria vector *Anopheles darlingi* in Central and South America. BMC Ecol.

[13] Marinotti O, Cerqueira GC, de Almeida LGP, Ferro MIT, da Loreto ELS, Zaha A (2013). The genome of *Anopheles darlingi*, the main neotropical malaria vector. Nucleic Acids Res..

[14] Zamora Perea E, Balta León R, Palomino Salcedo M, Brogdon WG, Devine GJ (2009). Adaptation and evaluation of the bottle assay for monitoring insecticide resistance in disease vector mosquitoes in the Peruvian Amazon. Malar J.

[15] Kobylinski KC, Escobedo-Vargas KS, López-Sifuentes VM, Durand S, Smith ES, Baldeviano GC (2017). Ivermectin susceptibility, sporontocidal effect, and inhibition of time to re-feed in the Amazonian malaria vector *Anopheles darlingi*. Malar J.

[16] Rocha EM, de Katak RM, Campos de Oliveira J, da Araujo MS, Carlos BC, Galizi R (2020). Vector-focused approaches to curb malaria transmission in the Brazilian Amazon: an overview of current and future challenges and strategies. Trop Med Infect Dis..

[17] Conn JE, Ribolla PE, Adelman ZN (2016). Chapter 5—Ecology of *Anopheles darlingi*, the primary malaria vector in the Americas and current nongenetic methods of vector control. Genetic Control of Malaria and Dengue.

[18] Baia-da-Silva DC, Brito-Sousa JD, Rodovalho SR, Peterka C, Moresco G, Lapouble OMM (2019). Current vector control challenges in the fight against malaria in Brazil. Rev Soc Bras Med Trop.

[19] Terenius O, Marinotti O, Sieglaff D, James AA (2008). Molecular genetic manipulation of vector mosquitoes. Cell Host Microbe.

[20] Carballar-Lejarazú R, James AA (2017). Population modification of Anopheline species to control malaria transmission. Pathog Glob Health.

[21] Wilke ABB, Marrelli MT (2015). Paratransgenesis: a promising new strategy for mosquito vector control. Parasit Vectors.

[22] Li J, Han M, Yu J (2018). Simple paratransgenic mosquitoes models and their dynamics. Math Biosci.

[23] Huang W, Wang S, Jacobs-Lorena M (2020). Use of microbiota to fight mosquito-borne disease. Front Genet.

[24] Asgari M, Ilbeigikhamsehnejad M, Rismani E, Dinparast Djadid N, Raz A (2020). Molecular characterization of RNase III protein of *Asaia sp*. for developing a robust RNAi-based paratransgensis tool to affect the sexual life-cycle of Plasmodium or Anopheles fitness. Parasit Vectors..

[25] Yoshida S, Ioka D, Matsuoka H, Endo H, Ishii A (2001). Bacteria expressing single-chain immunotoxin inhibit malaria parasite development in mosquitoes. Mol Biochem Parasitol.

[26] Wang S, Ghosh AK, Bongio N, Stebbings KA, Lampe DJ, Jacobs-Lorena M (2012). Fighting malaria with engineered symbiotic bacteria from vector mosquitoes. Proc Natl Acad Sci USA.

[27] Wang S, Dos-Santos ALA, Huang W, Liu KC, Oshaghi MA, Wei G (2017). Driving mosquito refractoriness to *Plasmodium falciparum* with engineered symbiotic bacteria. Science.

[28] Villegas LM, Pimenta PFP, Villegas LM, Pimenta PFP (2014). Metagenomics, paratransgenesis and the *Anopheles* microbiome: a portrait of the geographical distribution of the anopheline microbiota based on a meta-analysis of reported taxa. Mem Inst Oswaldo Cruz.

[29] Mancini MV, Spaccapelo R, Damiani C, Accoti A, Tallarita M, Petraglia E (2016). Paratransgenesis to control malaria vectors: a semi-field pilot study. Parasit Vectors.

[30] Raharimalala FN, Boukraa S, Bawin T, Boyer S, Francis F (2016). Molecular detection of six (endo-) symbiotic bacteria in Belgian mosquitoes: first step towards the selection of appropriate paratransgenesis candidates. Parasitol Res.

[31] Terenius O, de Oliveira CD, Pinheiro WD, Tadei WP, James AA, Marinotti O (2008). 16S rRNA Gene sequences from bacteria associated with adult *Anopheles darlingi* (Diptera: Culicidae) mosquitoes. J Med Entomol.

[32] Kämpfer P, Glaeser SP, Marinotti O, Guy L, Håkansson S, Tadei WP (2016). *Coetzeea brasiliensis* gen. nov., sp. Nov. isolated from larvae of Anopheles darlingi. Int J Syst Evol Microbiol..

[33] Arruda A, Ferreira GS, da Lima NCS, dos Santos Júnior A, Custódio MGF, Benevides-Matos N (2017). A simple methodology to collect culturable bacteria from feces of *Anopheles darlingi* (Diptera: Culicidae). J Microbiol Methods..

[34] Nilsson LKJ, de Oliveira MR, Marinotti O, Rocha EM, Håkansson S, Tadei WP (2019). Characterization of bacterial communities in breeding waters of *Anopheles darlingi* in Manaus in the Amazon Basin malaria-endemic area. Microb Ecol.

[35] Oliveira TMP, Sanabani SS, Sallum MAM (2020). Bacterial diversity associated with the abdomens of naturally *Plasmodium*-infected and non-infected *Nyssorhynchus darlingi*. BMC Microbiol.

[36] Oliveira TMP, Sanabani SS, Sallum MAM, Oliveira TMP, Sanabani SS, Sallum MAM (2020). *Asaia* (Rhodospirillales: Acetobacteraceae) and *Serratia* (Enterobacterales: Yersiniaceae) associated with *Nyssorhynchus braziliensis* and *Nyssorhynchus darlingi* (Diptera: Culicidae). Rev Bras Entomol.

[37] Agogué H, Casamayor EO, Joux F, Obernosterer I, Dupuy C, Lantoine F (2004). Comparison of samplers for the biological characterization of the sea surface microlayer. Limnol Oceanogr Methods.

[38] Service MW (1993). Mosquito ecology field sampling methods.

[39] Consoli RAGB, Oliveira RL de. Principais mosquitos de importância sanitária no Brasil. Editora FIOCRUZ; 1994.

[40] Fukatsu T, Nikoh N (1998). Two Intracellular Symbiotic Bacteria from the Mulberry Psyllid Anomoneura mori (Insecta, Homoptera). Appl Environ Microbiol.

[41] Sawada H, Ieki H, Oyaizu H, Matsumoto S (1993). Proposal for rejection of *Agrobacterium tumefaciens* and revised descriptions for the Genus *Agrobacterium* and for *Agrobacterium radiobacter* and *Agrobacterium rhizogenes*. Int J Syst Evol Microbiol.

[42] Huang X, Madan A (1999). CAP3: a DNA sequence assembly program. Genome Res.

[43] Gonzales MF, Brooks T, Pukatzki SU, Provenzano D (2013). Rapid protocol for preparation of electrocompetent *Escherichia coli* and *Vibrio cholerae*. J Vis Exp.

[44] Pumpuni CB, Beier MS, Nataro JP, Guers LD, Davis JR (1993). *Plasmodium falciparum*: Inhibition of sporogonic development in *Anopheles stephensi* by Gram-negative bacteria. Exp Parasitol.

[45] Dong Y, Manfredini F, Dimopoulos G (2009). Implication of the mosquito midgut microbiota in the defense against malaria parasites. PLoS Pathog.

[46] Gendrin M, Christophides GK. The *Anopheles* mosquito microbiota and their impact on pathogen transmission. In: *Anopheles* Mosquitoes—New Insights Malar Vectors. Manguin S, Ed. IntechOpen; 2013. https://www.intechopen.com/books/anopheles-mosquitoes-new-insights-into-malaria-vectors/the-anopheles-mosquito-microbiota-and-their-impact-on-pathogen-transmission.

[47] Boissière A, Tchioffo MT, Bachar D, Abate L, Marie A, Nsango SE (2012). Midgut microbiota of the malaria mosquito vector *Anopheles gambiae* and interactions with *Plasmodium falciparum* infection. PLoS Pathog.

[48] Chavshin AR, Oshaghi MA, Vatandoost H, Pourmand MR, Raeisi A, Terenius O (2014). Isolation and identification of culturable bacteria from wild *Anopheles culicifacies*, a first step in a paratransgenesis approach. Parasit Vectors.

[49] Ngo CT, Romano-Bertrand S, Manguin S, Jumas-Bilak E (2016). Diversity of the bacterial Microbiota of *Anopheles* mosquitoes from Binh Phuoc Province. Vietnam Front Microbiol.

[50] Rami A, Raz A, Zakeri S, Dinparast DN (2018). Isolation and identification of *Asaia* sp. in *Anopheles* spp. mosquitoes collected from Iranian malaria settings: steps toward applying paratransgenic tools against malaria. Parasit Vectors..

[51] Galeano-Castañeda Y, Urrea-Aguirre P, Piedrahita S, Bascuñán P, Correa MM (2019). Composition and structure of the culturable gut bacterial communities in *Anopheles albimanus* from Colombia. PLoS ONE.

[52] Berhanu A, Abera A, Nega D, Mekasha S, Fentaw S, Assefa A (2019). Isolation and identification of microflora from the midgut and salivary glands of *Anopheles* species in malaria endemic areas of Ethiopia. BMC Microbiol.

[53] Seibert G, Hörner R, Meneghetti BH, Righi RA, Forno NLFD, Salla A, et al. Nosocomial infections by *Klebsiella pneumoniae* carbapenemase producing enterobacteria in a teaching hospital. Einstein São Paulo. Instituto Israelita de Ensino e Pesquisa Albert Einstein; 2014;12:282–6. 10.1590/S1679-45082014AO3131 PMC4872936 25295446

[54] Subhadra B, Surendran S, Lim BR, Yim J-S, Kim DH, Woo K (2019). Complete genome sequence and phylogenetic analysis of nosocomial pathogen *Acinetobacter nosocomialis* strain NCTC 8102. Genes Genomics.

[55] Rani A, Sharma A, Rajagopal R, Adak T, Bhatnagar RK (2009). Bacterial diversity analysis of larvae and adult midgut microflora using culture-dependent and culture-independent methods in lab-reared and field-collected *Anopheles stephensi*-an Asian malarial vector. BMC Microbiol.

[56] Crotti E, Rizzi A, Chouaia B, Ricci I, Favia G, Alma A (2010). Acetic acid bacteria, newly emerging symbionts of insects. Appl Environ Microbiol.

[57] Binetruy F, Dupraz M, Buysse M, Duron O (2019). Surface sterilization methods impact measures of internal microbial diversity in ticks. Parasit Vectors.

[58] Ribeiro MM, Neumann VA, Padoveze MC, Graziano KU, Ribeiro MM, Neumann VA (2015). Eficacia y efectividad del alcohol en la desinfección de materiales semicríticos: revisión sistemática. Rev Lat Am Enfermagem.

[59] Moll RM, Romoser WS, Modrakowski MC, Moncayo AC, Lerdthusnee K (2001). Meconial peritrophic membranes and the fate of midgut bacteria during mosquito (Diptera: Culicidae) metamorphosis. J Med Entomol.

[60] Cifelli AN. Horizontal and vertical transmission of a *Pantoea* Sp. in *Culex* Sp. [Thesis]. 2015. http://dspace.calstate.edu/handle/10211.3/196892.

[61] Koosha M, Vatandoost H, Karimian F, Choubdar N, Oshaghi MA (2019). Delivery of a genetically marked Serratia AS1 to medically important arthropods for use in RNAi and paratransgenic control strategies. Microb Ecol.

