# Correction to: Culturable bacteria associated with Anopheles darlingi and their paratransgenesis potential

**DOI:** 10.1186/s12936-021-03609-1

**Published:** 2021-02-10

**Authors:** Elerson Matos Rocha, Osvaldo Marinotti, Deidre Machado Serrão, Laura Viana Correa, Ricardo de Melo Katak, Juan Campos de Oliveira, Veranilce Alves Muniz, Marta Rodrigues de Oliveira, Joaquim Ferreira do Nascimento Neto, Marcos Cézar Fernandes Pessoa, Rosemary Aparecida Roque, Adolfo Jose da Mota, Piero Onorati, Jayme A. Souza-Neto, Olle Terenius, Wanderli Pedro Tadei

**Affiliations:** 1 Programa de Pós-Graduação em Biotecnologia, Universidade Federal do Amazonas - PPGBIOTEC/UFAM, Manaus, Brazil; 2 MTEKPrime, Aliso Viejo, CA USA; 3 Universidade Estadual do Amazonas - MBT, UEA, Manaus, Brazil; 4 Universidade Estadual do Amazonas - BIONORTE, UEA, Manaus, Brazil; 5 Laboratório de Malária E Dengue, Instituto Nacional de Pesquisas da Amazônia, INPA, Manaus, Brazil; 6 Department of Ecology, Swedish University of Agricultural Sciences (SLU), Box 7044, 750 07 Uppsala, Sweden; 7 School of Agricultural Sciences, Department of Bioprocesses and Biotechnology, Central Multi User Laboratory, São Paulo State University (UNESP), Botucatu, Brazil; 8 Department of Cell and Molecular Biology, Microbiology, Uppsala University, Box 596, 751 24 Uppsala, Sweden

## Correction to: Malar J (2021) 20:40 10.1186/s12936-020-03574-1

Following publication of the original article [1], it came to the authors’ attention that the article had published with two author names misspelled: ‘Juan Campos de Oliveira’ had been written as ‘Campos-de-Oliveir Juan’, while ‘Joaquim Ferreira do Nascimento Neto’ had been written as ‘Joaquim Ferreira’.

Please find the corrected names in the author list of this correction.

The names have since been corrected in the published article.
