# Supplementary material for: Culturable bacteria associated with Anopheles darlingi and their paratransgenesis potential
Source: Malar J. 2021 Jan 13;20:40. doi: 10.1186/s12936-020-03574-1 (PMC7805163; doi:10.1186/s12936-020-03574-1)
Supplement: Supplementary file 1 — Additional file 1: Table S1. Bacteria isolated from samples from Coari. Table S2. Proteobacteria isolated from samples from Manaus. Table S3. Firmicutes isolated from samples from Manaus. Table S4. Bacteroidetes isolated from samples from Manaus. Table S5. Actinobacteria isolated from samples from Manaus. [file 12936_2020_3574_MOESM1_ESM.docx]

**Additional material**

Additional document 1

pSPT-1-GFP sequence

>pSPT-1-GFP

CTAAATTGTAAGCGTTAATATTTTGTTAAAATTCGCGTTAAATTTTTGTTAAATCAGCTCATTTTTTAACCAATAGGCCGAAATCGGCAAAATCCCTTATAAATCAAAAGAATAGACCGAGATAGGGTTGAGTGTTGTTCCAGTTTGGAACAAGAGTCCACTATTAAAGAACGTGGACTCCAACGTCAAAGGGCGAAAAACCGTCTATCAGGGCGATGGCCCACTACGTGAACCATCACCCTAATCAAGTTTTTTGGGGTCGAGGTGCCGTAAAGCACTAAATCGGAACCCTAAAGGGAGCCCCCGATTTAGAGCTTGACGGGGAAAGCCGGCGAACGTGGCGAGAAAGGAAGGGAAGAAAGCGAAAGGAGCGGGCGCTAGGGCGCTGGCAAGTGTAGCGGTCACGCTGCGCGTAACCACCACACCCGCCGCGCTTAATGCGCCGCTACAGGGCGCGTCCCATTCGCCATTCAGGCTGCGCAACTGTTGGGAAGGGCGATCGGTGCGGGCCTCTTCGCTATTACGCCAGCTGGCGAAAGGGGGATGTGCTGCAAGGCGATTAAGTTGGGTAACGCCAGGGTTTTCCCAGTCACGACGTTGTAAAACGACGGCCAGTGAGCGCGCGTAATACGACTCACTATAGGGCGAATTGGCGGCCGCAATTTCTTGACAAATTCTTAAATTTGTGCTATAATGTATCGAATTGTGAGCGCTCACAATTATAATAATTTTGTTTAACTTTAAGAAGGAGATATACATATGGCTAGCAAAGGAGAAGAACTTTTCACTGGAGTTGTCCCAATTCTTGTTGAATTAGATGGTGATGTTAATGGGCACAAATTTTCTGTCAGTGGAGAGGGTGAAGGTGATGCTACATACGGAAAACTTACCCTTAAATTTATTTGCACTACTGGAAAACTACCTGTTCCATGGCCAACACTTGTCACTACTTTCTCTTATGGTGTTCAATGCTTTTCCCGTTATCCGGATCACATGAAACGGCATGACTTTTTCAAGAGTGCCATGCCCGAAGGTTATGTACAGGAACGCACTATATCTTTCAAAGATGACGGGAACTACAAGACGCGTGCTGAAGTCAAGTTTGAAGGTGATACCCTTGTTAATCGTATCGAGTTAAAAGGTATTGATTTTAAAGAAGATGGAAACATTCTCGGACACAAACTGGAGTACAACTATAACTCACACAATGTATACATCACGGCAGACAAACAAAAGAATGGAATCAAAGCTAACTTCAAAATTCGCCACAACATTGAAGATGGCTCCGTTCAACTAGCAGACCATTATCAACAAAATACTCCAATTGGCGATGGCCCTGTCCTTTTACCAGACAACCATTACCTGTCTACACAATCTGCCCTTTCGAAAGATCCCAACGAAAAGCGTGACCACATGGTCCTTCTTGAGTTTGTAACTGCTGCTGGGATTACACATGGCATGGATGAGCTCTACAAATAAGGATCCATCGGAAGCCCGCCTAATGAGCGGGCTTTTTTTTAAGCTAGCGGCCGCCAGCTTTTGTTCCCTTTAGTGAGGGTTAATTGCGCGCTTGGCGTAATCATGGTCATAGCTGTTTCCTGTGTGAAATTGTTATCCGCTCACAATTCCACACAACATACGAGCCGGAAGCATAAAGTGTAAAGCCTGGGGTGCCTAATGAGTGAGCTAACTCACATTAATTGCGTTGCGCTCACTGCCCGCTTTCCAGTCGGGAAACCTGTCGTGCCAGCTGCATTAATGAATCGGCCAACGCGCGGGGAGAGGCGGTTTGCGTATTGGGCGCTCTTCCGCTTCCTCGCTCACTGACTCGCTGCGCTCGGTCGTTCGGCTGCGGCGAGCGGTATCAGCTCACTCAAAGGCGGTAATACGGTTATCCACAGAATCAGGGGATAACGCAGGAAAGAACATGTGAGCAAAAGGCCAGCAAAAGGCCAGGAACCGTAAAAAGGCCGCGTTGCTGGCGTTTTTCCATAGGCTCCGCCCCCCTGACGAGCATCACAAAAATCGACGCTCAAGTCAGAGGTGGCGAAACCCGACAGGACTATAAAGATACCAGGCGTTTCCCCCTGGAAGCTCCCTCGTGCGCTCTCCTGTTCCGACCCTGCCGCTTACCGGATACCTGTCCGCCTTTCTCCCTTCGGGAAGCGTGGCGCTTTCTCATAGCTCACGCTGTAGGTATCTCAGTTCGGTGTAGGTCGTTCGCTCCAAGCTGGGCTGTGTGCACGAACCCCCCGTTCAGCCCGACCGCTGCGCCTTATCCGGTAACTATCGTCTTGAGTCCAACCCGGTAAGACACGACTTATCGCCACTGGCAGCAGCCACTGGTAACAGGATTAGCAGAGCGAGGTATGTAGGCGGTGCTACAGAGTTCTTGAAGTGGTGGCCTAACTACGGCTACACTAGAAGGACAGTATTTGGTATCTGCGCTCTGCTGAAGCCAGTTACCTTCGGAAAAAGAGTTGGTAGCTCTTGATCCGGCAAACAAACCACCGCTGGTAGCGGTGGTTTTTTTGTTTGCAAGCAGCAGATTACGCGCAGAAAAAAAGGATCTCAAGAAGATCCTTTGATCTTTTCTACGGGGTCTGACGCTCAGTGGAACGAAAACTCACGTTAAGGGATTTTGGTCATGAGATTATCAAAAAGGATCTTCACCTAGATCCTTTTAAATTAAAAATGAAGTTTTAAATCAATCTAAAGTATATATGAGTAAACTTGGTCTGACAGTTACCAATGCTTAATCAGTGAGGCACCTATCTCAGCGATCTGTCTATTTCGTTCATCCATAGTTGCCTGACTCCCCGTCGTGTAGATAACTACGATACGGGAGGGCTTACCATCTGGCCCCAGTGCTGCAATGATACCGCGAGACCCACGCTCACCGGCTCCAGATTTATCAGCAATAAACCAGCCAGCCGGAAGGGCCGAGCGCAGAAGTGGTCCTGCAACTTTATCCGCCTCCATCCAGTCTATTAATTGTTGCCGGGAAGCTAGAGTAAGTAGTTCGCCAGTTAATAGTTTGCGCAACGTTGTTGCCATTGCTACAGGCATCGTGGTGTCACGCTCGTCGTTTGGTATGGCTTCATTCAGCTCCGGTTCCCAACGATCAAGGCGAGTTACATGATCCCCCATGTTGTGCAAAAAAGCGGTTAGCTCCTTCGGTCCTCCGATCGTTGTCAGAAGTAAGTTGGCCGCAGTGTTATCACTCATGGTTATGGCAGCACTGCATAATTCTCTTACTGTCATGCCATCCGTAAGATGCTTTTCTGTGACTGGTGAGTACTCAACCAAGTCATTCTGAGAATAGTGTATGCGGCGACCGAGTTGCTCTTGCCCGGCGTCAATACGGGATAATACCGCGCCACATAGCAGAACTTTAAAAGTGCTCATCATTGGAAAACGTTCTTCGGGGCGAAAACTCTCAAGGATCTTACCGCTGTTGAGATCCAGTTCGATGTAACCCACTCGTGCACCCAACTGATCTTCAGCATCTTTTACTTTCACCAGCGTTTCTGGGTGAGCAAAAACAGGAAGGCAAAATGCCGCAAAAAAGGGAATAAGGGCGACACGGAAATGTTGAATACTCATACTCTTCCTTTTTCAATATTATTGAAGCATTTATCAGGGTTATTGTCTCATGAGCGGATACATATTTGAATGTATTTAGAAAAATAAACAAATAGGGGTTCCGCGCACATTTCCCCGAAAAGTGCCAC

**Additional Tables**

**Table S1:** Bacteria isolated from samples from Coari

| **Phylum** | **Strain** | **Family** | **Genus/Species** | **% identity** | **Gram** | **GenBank NCBI accession number** |
| --- | --- | --- | --- | --- | --- | --- |
| Proteobacteria | C41 | Moraxellaceae | *Acinetobacter* sp | 100% | Coccobacilli - | MT052388 |
|  | C61 | Alcaligenaceae | *Achromobacter* sp | 100% | Bacilli - | MT052393 |
|  | C64 | Alcaligenaceae | *Achromobacter* sp | 100% | Bacilli - | MT052394 |
|  | C30 | Enterobacteriaceae | *Klebsiella* sp | 100% | Bacilli - | MT052383 |
|  | C6 | Yersiniaceae | *Serratia sp* | 100% | Bacilli - | MT052375 |
|  | C9 | Yersiniaceae | *Serratia* sp | 100% | Bacilli - | MT052377 |
|  | C19 | Yersiniaceae | *Serratia* sp | 100% | Bacilli - | MT052379 |
|  | C36 | Yersiniaceae | *Serratia* sp | 100% | Bacilli - | MT052384 |
|  | C37 | Yersiniaceae | *Serratia marcescens* | 99% | Bacilli - | MT052385 |
|  | C38 | Yersiniaceae | *Serratia* sp | 100% | Bacilli - | MT052386 |
|  | C58 | Yersiniaceae | *Serratia* sp | 100% | Bacilli - | MT052392 |
|  | C5 | Enterobacteriaceae | *Siccibacter* sp | 100% | Bacilli - | MT052374 |
|  | C7 | Enterobacteriaceae | *Siccibacter* sp | 100% | Bacilli - | MT052376 |
|  | C29 | Enterobacteriaceae | *Siccibacter* sp | 100% | Bacilli + | MT052382 |
| Firmicutes | C14 | Bacillaceae | *Bacillus* sp | 100% | Bacilli + | MT052378 |
|  | C55 | Bacillaceae | *Bacillus* sp | 100% | Bacilli + | MT052391 |
|  | C4 | Bacillaceae | *Bacillus* sp | 100% | Bacilli + | MT052373 |
|  | C67 | Bacillaceae | *Bacillus sp* | 100% | Bacilli + | MT052395 |
|  | C2 | Bacillaceae | *Bacillus sp* | 100% | Bacilli + | MT052372 |
|  | C21 | Bacillaceae | *Bacillus* sp | 100% | Bacilli + | MT052380 |
|  | C39 | Bacillaceae | *Bacillus* sp | 100% | Bacilli + | MT052387 |
|  | C22 | Bacillaceae | *Bacillus* sp | 100% | Bacilli + | MT052381 |
|  | C45 | Bacillaceae | *Bacillus* sp | 100% | Bacilli + | MT052389 |
|  | C46 | Bacillaceae | *Bacillus* sp | 100% | Bacilli + | MT052390 |

Taxonomic assignments of the isolated bacteria based on 16S ribosomal RNA gene, shape and Gram staining. Percentage identity indicates identity with the best match within the NCBI database. GenBank accession numbers for the 16S ribosomal RNA gene sequences of all strains isolated in this study are provided in the last column.

**Table S2:** Proteobacteria isolated from samples from Manaus

| **Strain** | **Family** | **Genus/Species** | **% identity** | **Gram** | **GenBank NCBI**  **accession number** |
| --- | --- | --- | --- | --- | --- |
| Ovo9 | Moraxellaceae | *Acinetobacter* sp | 100% | Coccobacilli - | MN709229 |
| Adu2 | Moraxellaceae | *Acinetobacter* sp | 100% | Coccobacilli - | MN709294 |
| Wat25 | Moraxellaceae | *Acinetobacter* sp | 100% | Coccobacilli - | MN709366 |
| Ovo14 | Moraxellaceae | *Acinetobacter* sp | 100% | Coccobacilli - | MN709234 |
| Lar11 | Moraxellaceae | *Acinetobacter* sp | 100% | Coccobacilli - | MN709252 |
| Adu22 | Moraxellaceae | *Acinetobacter* sp | 100% | Bacilli - | MN709314 |
| Pup5 | Moraxellaceae | *Acinetobacter* sp | 100% | Coccobacilli - | MN709274 |
| Adu29 | Moraxellaceae | *Acinetobacter* sp | 100% | Coccobacilli - | MN709321 |
| Lar6 | Moraxellaceae | *Acinetobacter* sp | 100% | Coccobacilli - | MN709247 |
| Adu16 | Moraxellaceae | *Acinetobacter nosocomialis* | 100% | Coccobacilli - | MN709308 |
| Pup14 | Moraxellaceae | *Acinetobacter nosocomialis* | 100% | Coccobacilli - | MN709283 |
| Adu44 | Moraxellaceae | *Acinetobacter nosocomialis* | 100% | Coccobacilli - | MN709336 |
| Wat1 | Moraxellaceae | *Acinetobacter* sp | 100% | Coccobacilli - | MN709342 |
| Lar3 | Moraxellaceae | *Acinetobacter* sp | 100% | Coccobacilli - | MN709244 |
| Adu48 | Moraxellaceae | *Acinetobacter nosocomialis* | 99% | Coccobacilli - | MN709340 |
| Wat3 | Moraxellaceae | *Acinetobacter* sp | 100% | Coccobacilli - | MN709344 |
| Lar27 | Moraxellaceae | *Acinetobacter* sp | 100% | Coccobacilli - | MN709268 |
| Wat5 | Moraxellaceae | *Acinetobacter* sp | 99% | Coccobacilli - | MN709346 |
| Adu6 | Moraxellaceae | *Acinetobacter nosocomialis* | 99% | Coccobacilli - | MN709298 |
| Wat15 | Moraxellaceae | *Acinetobacter* sp | 100% | Coccobacilli - | MN709356 |
| Lar15 | Moraxellaceae | *Acinetobacter nosocomialis* | 99% | Coccobacilli - | MN709256 |
| Wat21 | Moraxellaceae | *Acinetobacter nosocomialis* | 99% | Coccobacilli - | MN709362 |
| Wat27 | Moraxellaceae | *Acinetobacter nosocomialis* | 99% | Coccobacilli - | MN709368 |
| Lar19 | Moraxellaceae | *Acinetobacter* sp | 99% | Coccobacilli - | MN709260 |
| Adu21 | Moraxellaceae | *Acinetobacter* sp | 99% | Coccobacilli - | MN709313 |
| Pup19 | Moraxellaceae | *Acinetobacter seifertii* | 100% | Coccobacilli - | MN709288 |
| Adu36 | Moraxellaceae | *Acinetobacter* sp | 100% | Bacilli - | MN709328 |
| Pup4 | Aeromonadaceae | *Aeromonas caviae* | 100% | Bacilli - | MN709273 |
| Adu13 | Aeromonadaceae | *Aeromonas* sp | 100% | Bacilli - | MN709305 |
| Ovo19 | Chromobacteriaceae | *Aquitalea* sp | 99% | Bacilli - | MN709239 |
| Adu45 | Chromobacteriaceae | *Aquitalea* sp | 100% | Bacilli - | MN709337 |
| Pup15 | Rhodospirillaceae | *Azospirillum* sp | 100% | Bacilli - | MN709284 |
| Lar10 | Burkholderiaceae | *Burkholderia* sp | 100% | Bacilli - | MN709251 |
| Wat4 | Chromobacteriaceae | *Chromobacterium* sp | 100% | Bacilli - | MN709345 |
| Adu7 | Chromobacteriaceae | *Chromobacterium* sp | 100% | Bacilli - | MN709299 |
| Pup6 | Chromobacteriaceae | *Chromobacterium* sp | 100% | Bacilli - | MN709275 |
| Adu23 | Chromobacteriaceae | *Chromobacterium* sp | 100% | Bacilli - | MN709315 |
| Wat16 | Chromobacteriaceae | *Chromobacterium* sp | 100% | Bacilli - | MN709357 |
| Lar4 | Chromobacteriaceae | *Chromobacterium* sp | 100% | Bacilli - | MN709245 |
| Wat26 | Chromobacteriaceae | *Chromobacterium* sp | 100% | Bacilli - | MN709367 |
| Adu18 | Weeksellaceae | *Chryseobacterium* sp | 100% | Bacilli - | MN709310 |
| Adu1 | Weeksellaceae | *Chryseobacterium* sp | 100% | Bacilli - | MN709293 |
| Wat12 | Weeksellaceae | *Chryseobacterium* sp | 100% | Bacilli - | MN709353 |
| Adu28 | Weeksellaceae | *Chryseobacterium* sp | 100% | Bacilli - | MN709320 |
| Adu26 | Weeksellaceae | *Chryseobacterium* sp | 100% | Bacilli - | MN709318 |
| Adu37 | Weeksellaceae | *Chryseobacterium* sp | 100% | Bacilli - | MN709329 |
| Lar7 | Weeksellaceae | *Chryseobacterium* sp | 100% | Bacilli - | MN709248 |
| Wat20 | Weeksellaceae | *Chryseobacterium* sp | 100% | Bacilli - | MN709361 |
| Wat22 | Weeksellaceae | *Chryseobacterium* sp | 100% | Bacilli - | MN709363 |
| Ovo1 | Enterobacteriaceae | *Citrobacter* sp | 99% | Bacilli - | MN709221 |
| Lar26 | Enterobacteriaceae | *Cronobacter* sp | 99% | Bacilli - | MN709267 |
| Wat34 | Burkholderiaceae | *Cupriavidus* sp | 100% | Bacilli - | MN709375 |
| Ovo6 | Burkholderiaceae | *Cupriavidus* sp | 100% | Bacilli - | MN709226 |
| Adu8 | Burkholderiaceae | *Cupriavidus* sp | 100% | Bacilli - | MN709300 |
| Adu17 | Burkholderiaceae | *Cupriavidus* sp | 100% | Bacilli - | MN709309 |
| Lar9 | Enterobacteriaceae | *Enterobacter* sp | 100% | Bacilli - | MN709250 |
| Adu30 | Enterobacteriaceae | *Enterobacter* sp | 100% | Bacilli - | MN709322 |
| Pup17 | Enterobacteriaceae | *Enterobacter asburiae* | 100% | Bacilli - | MN709286 |
| Wat19 | Enterobacteriaceae | *Enterobacter* sp | 100% | Bacilli - | MN709360 |
| Adu24 | Enterobacteriaceae | *Enterobacter asburiae* | 99% | Bacilli - | MN709316 |
| Ovo2 | Enterobacteriaceae | *Enterobacter asburiae* | 99% | Bacilli - | MN709222 |
| Lar16 | Enterobacteriaceae | *Enterobacter asburiae* | 99% | Bacilli - | MN709257 |
| Adu49 | Enterobacteriaceae | *Enterobacter* sp | 100% | Bacilli - | MN709341 |
| Wat6 | Enterobacteriaceae | [*Enterobacter asburiae*](https://blast.ncbi.nlm.nih.gov/Blast.cgi#alnHdr_1540583396) | 100% | Bacilli - | MN709347 |
| Adu20 | Enterobacteriaceae | *Enterobacter* sp | 100% | Bacilli - | MN709312 |
| Lar12 | Enterobacteriaceae | *Enterobacter* sp | 100% | Bacilli - | MN709253 |
| Wat7 | Enterobacteriaceae | *Enterobacter* sp | 100% | Bacilli - | MN709348 |
| Wat13 | Enterobacteriaceae | *Enterobacter* sp | 100% | Bacilli - | MN709354 |
| Lar24 | Enterobacteriaceae | *Enterobacter* sp | 100% | Bacilli - | MN709265 |
| Adu31 | Oxalobacteraceae | *Herbaspirillum* sp | 100% | Bacilli - | MN709323 |
| Lar5 | Enterobacteriaceae | *Klebsiella* sp | 100% | Bacilli - | MN709246 |
| Adu14 | Enterobacteriaceae | *Klebsiella* sp | 100% | Bacilli - | MN709306 |
| Wat8 | Enterobacteriaceae | *Klebsiella* sp | 100% | Bacilli - | MN709349 |
| Wat14 | Enterobacteriaceae | *Klebsiella* sp | 100% | Bacilli - | MN709355 |
| Lar25 | Enterobacteriaceae | *Klebsiella* sp | 100% | Bacilli - | MN709266 |
| Wat24 | Enterobacteriaceae | *Klebsiella* sp | 100% | Bacilli - | MN709365 |
| Ovo7 | Enterobacteriaceae | *Klebsiella* sp | 100% | Bacilli - | MN709227 |
| Wat33 | Enterobacteriaceae | *Klebsiella* sp | 100% | Bacilli - | MN709374 |
| Pup18 | Enterobacteriaceae | *Klebsiella* sp | 100% | Bacilli - | MN709287 |
| Adu47 | Enterobacteriaceae | *Klebsiella* sp | 100% | Bacilli - | MN709339 |
| Adu35 | Enterobacteriaceae | *Klebsiella variicola* | 99% | Bacilli - | MN709327 |
| Pup20 | Enterobacteriaceae | *Klebsiella variicola* | 100% | Bacilli - | MN709289 |
| Wat2 | Enterobacteriaceae | *Klebsiella* sp | 100% | Bacilli - | MN709343 |
| Wat28 | Enterobacteriaceae | *Klebsiella* sp | 100% | Bacilli - | MN709369 |
| Adu9 | Moraxellaceae | *Moraxella* sp | 100% | Cocci - | MN709301 |
| Ovo8 | Moraxellaceae | *Moraxella* sp | 100% | Cocci - | MN709228 |
| Ovo3 | Erwiniaceae | *Pantoea* sp | 99% | Bacilli - | MN709223 |
| Wat32 | Erwiniaceae | *Pantoea* sp | 100% | Bacilli - | MN709373 |
| Pup12 | Erwiniaceae | *Pantoea* sp | 99% | Bacilli - | MN709281 |
| Adu38 | Erwiniaceae | *Pantoea* sp | 100% | Bacilli - | MN709330 |
| Ovo15 | Erwiniaceae | *Pantoea* sp | 100% | Bacilli - | MN709235 |
| Lar13 | Erwiniaceae | *Pantoea* sp | 100% | Bacilli - | MN709254 |
| Adu3 | Erwiniaceae | *Pantoea* sp | 99% | Bacilli - | MN709295 |
| Pup11 | Pectobacteriaceae | *Pectobacterium* sp | 99% | Bacilli - | MN709280 |
| Wat31 | Pseudomononaceae | *Pseudomonas* sp | 100% | Bacilli - | MN709372 |
| Adu5 | Pseudomononaceae | *Pseudomonas* sp | 100% | Bacilli - | MN709297 |
| Ovo10 | Pseudomononaceae | *Pseudomonas* sp | 100% | Bacilli - | MN709230 |
| Adu46 | Pseudomononaceae | *Pseudomonas* sp | 100% | Bacilli - | MN709338 |
| Adu10 | Pseudomononaceae | *Pseudomonas* sp | 100% | Bacilli - | MN709302 |
| Lar17 | Pseudomononaceae | *Pseudomonas* sp | 99% | Bacilli - | MN709258 |
| Pup9 | Burkholderiaceae | *Ralstonia* sp | 100% | Bacilli - | MN709278 |
| Wat17 | Rhizobiaceae | *Rhizobium* sp | 100% | Bacilli - | MN709358 |
| Pup23 | Yersiniaceae | *Serratia* sp | 100% | Bacilli - | MN709292 |
| Adu40 | Yersiniaceae | *Serratia* sp | 100% | Bacilli - | MN709332 |
| Wat9 | Yersiniaceae | *Serratia* s*p* | 100% | Bacilli - | MN709350 |
| Wat23 | Yersiniaceae | *Serratia* sp | 100% | Bacilli - | MN709364 |
| Ovo4 | Yersiniaceae | *Serratia* sp | 100% | Bacilli - | MN709224 |
| Lar23 | Yersiniaceae | *Serratia* sp | 100% | Bacilli - | MN709264 |
| Lar14 | Yersiniaceae | *Serratia* sp | 100% | Bacilli - | MN709255 |
| Pup13 | Yersiniaceae | *Serratia* sp | 100% | Bacilli - | MN709282 |
| Wat11 | Enterobacteriaceae | *Stenotrophomonas* sp | 100% | Bacilli - | MN709352 |
| Ovo18 | Enterobacteriaceae | *Stenotrophomonas* sp | 100% | Bacilli - | MN709238 |
| Adu32 | Enterobacteriaceae | *Stenotrophomonas* sp | 100% | Bacilli - | MN709324 |
| Adu4 | Enterobacteriaceae | *Stenotrophomonas* sp | 100% | Bacilli - | MN709296 |

Taxonomic assignments of the isolated bacteria based on 16S ribosomal RNA gene, shape and Gram staining. Percentage identity indicates identity with the best match within the NCBI database. GenBank accession numbers for the 16S ribosomal RNA gene sequences of all strains isolated in this study are provided in the last column. Strains with names initiated with Ovo indicates they were isolated from eggs, similarly, Lar= larvae, Pup=pupae, Adu=adults and Wat=breeding site water.

**Table S3:** Firmicutes isolated from samples from Manaus

| **Strain** | **Family** | **Genus/Species** | **% identity** | **Gram** | **GenBank accession number** |
| --- | --- | --- | --- | --- | --- |
| Ovo20 | Bacillaceae | *Bacillus* sp | 100% | Bacilli + | MN709240 |
| Lar2 | Bacillaceae | *Bacillus* sp | 100% | Bacilli + | MN709243 |
| Ovo5 | Bacillaceae | *Bacillus* sp | 100% | Bacilli + | MN709225 |
| Adu39 | Bacillaeceae | *Bacillus* sp | 100% | Bacilli + | MN709331 |
| Pup2 | Bacillaceae | *Bacillus sp* | 100% | Bacilli + | MN709271 |
| Adu11 | Bacillaceae | *Bacillus sp* | 100% | Bacilli + | MN709303 |
| Adu25 | Bacillaceae | *Bacillus sp* | 100% | Bacilli + | MN709317 |
| Wat10 | Bacilaceae | *Bacillus sp* | 100% | Bacilli + | MN709351 |
| Ovo11 | Bacillaceae | *Bacillus sp* | 100% | Bacilli + | MN709231 |
| Pup10 | Bacillaceae | *Bacillus sp* | 100% | Bacilli + | MN709279 |
| Wat30 | Bacillaceae | *Bacillus* sp | 100% | Bacilli + | MN709371 |
| Lar22 | Bacillaceae | *Bacillus* sp | 100% | Bacilli + | MN709263 |
| Wat29 | Bacillaceae | *Bacillus* sp | 100% | Bacilli + | MN709370 |
| Pup22 | Bacillaceae | *Bacillus megaterium* | 100% | Bacilli + | MN709291 |
| Wat18 | Bacillaceae | *Bacillus* sp | 100% | Bacilli + | MN709359 |
| Pup1 | Bacillaceae | *Bacillus megaterium* | 100% | Bacilli + | MN709270 |
| Lar1 | Bacillaceae | *Bacillus* sp | 100% | Bacilli + | MN709242 |
| Adu43 | Bacillaceae | *Bacillus* sp | 100% | Bacilli + | MN709335 |
| Adu15 | Bacillaceae | *Bacillus* sp | 100% | Bacilli + | MN709307 |
| Pup7 | Bacillaceae | *Bacillus* sp | 100% | Bacilli + | MN709276 |
| Adu27 | Bacillaceae | *Bacillus sp* | 100% | Bacilli + | MN709319 |
| Lar28 | Bacillaceae | *Bacillus* sp | 100% | Bacilli + | MN709269 |
| Pup16 | Bacillaceae | *Exiguobacterium* sp | 100% | Bacilli + | MN709285 |
| Pup3 | Bacillaceae | *Flectobacillus* sp | 100% | Bacilli + | MN709272 |
| Adu41 | Bacillaceae | *Lysinibacillus* sp | 100% | Bacilli + | MN709333 |
| Ovo16 | Bacillaceae | *Lysinibacillus* sp | 100% | Bacilli + | MN709236 |
| Lar8 | Paenibacillaceae | *Paenibacillus* sp | 100% | Bacilli + | MN709249 |
| Adu33 | Staphylococcaceae | *Staphylococcus* sp | 100% | Cocci + | MN709325 |

Taxonomic assignments of the isolated bacteria based on 16S ribosomal RNA gene, shape and Gram staining. Percentage identity indicates identity with the best match within the NCBI database. GenBank accession numbers for the 16S ribosomal RNA gene sequences of all strains isolated in this study are provided in the last column. Strains with names initiated with Ovo indicates they were isolated from eggs, similarly, Lar= larvae, Pup=pupae, Adu=adults and Wat=breeding site water.

**Table S4:** Bacteroidetes isolated from samples from Manaus

| **Strain** | **Family** | **Species** | **% identity** | **Gram** | **GenBank accession number** |
| --- | --- | --- | --- | --- | --- |
| Ovo12 | Weeksellaceae | *Elizabethkingia* sp | 100% | Bacilli - | MN709232 |
| Adu12 | Weeksellaceae | *Elizabethkingia* sp | 100% | Bacilli - | MN709304 |
| Adu42 | Weeksellaceae | *Elizabethkingia* sp | 100% | Bacilli - | MN709334 |
| Lar21 | Weeksellaceae | *Elizabethkingia* sp | 100% | Bacilli - | MN709262 |
| Lar18 | Sphingobacteriaceae | *Nubsella* sp | 100% | Bacilli - | MN709259 |
| Ovo21 | Sphingobacteriaceae | *Sphingobacterium* sp | 100% | Bacilli - | MN709241 |

Taxonomic assignments of the isolated bacteria based on 16S ribosomal RNA gene, shape and Gram staining. Percentage identity indicates identity with the best match within the NCBI database. GenBank accession numbers for the 16S ribosomal RNA gene sequences of all strains isolated in this study are provided in the last column. Strains with names initiated with Ovo indicates they were isolated from eggs, similarly, Lar= larvae, Pup=pupae, Adu=adults and Wat=breeding site water.

**Table S5:** Actinobacteria isolated from samples from Manaus

| **Strain** | **Family** | **Species** | **% Identity** | **Gram** | **GenBank accession number** |
| --- | --- | --- | --- | --- | --- |
| Adu34 | Micrococcaceae | *Arthrobacter* sp | 100% | Bacilli + | MN709326 |
| Lar20 | Brevibacteriaceae | *Brevibacterium* sp | 100% | Bacilli + | MN709261 |
| Pup8 | Microbacteriaceae | *Leucobacter* sp | 100% | Bacilli + | MN709277 |
| Pup21 | Microbacteriaceae | *Leucobacter* sp | 100% | Bacilli + | MN709290 |
| Ovo13 | Microbacteriaceae | *Microbacterium* sp | 100% | Bacilli + | MN709233 |
| Adu19 | Microbacteriaceae | *Microbacterium* sp | 100% | Bacilli + | MN709311 |
| Ovo17 | Microbacteriaceae | *Microbacterium* sp | 100% | Bacilli + | MN709237 |

Taxonomic assignments of the isolated bacteria based on 16S ribosomal RNA gene, shape and Gram staining. Percentage identity indicates identity with the best match within the NCBI database. GenBank accession numbers for the 16S ribosomal RNA gene sequences of all strains isolated in this study are provided in the last column. Strains with names initiated with Ovo indicates they were isolated from eggs, similarly, Lar= larvae, Pup=pupae, Adu=adults and Wat=breeding site water.
